# The Relationship between the Level of Anterior Cingulate Cortex Metabolites, Brain-Periphery Redox Imbalance, and the Clinical State of Patients with Schizophrenia and Personality Disorders

**DOI:** 10.3390/biom10091272

**Published:** 2020-09-03

**Authors:** Amira Bryll, Wirginia Krzyściak, Paulina Karcz, Natalia Śmierciak, Tamas Kozicz, Justyna Skrzypek, Marta Szwajca, Maciej Pilecki, Tadeusz J. Popiela

**Affiliations:** 1 Department of Radiology, Jagiellonian University Medical College, Kopernika 19, 31-501 Krakow, Poland; amira.bryll@uj.edu.pl; 2 Department of Medical Diagnostics, Jagiellonian University, Medical College, Medyczna 9, 30-688 Krakow, Poland; justynaskrzypek96@gmail.com; 3 Department of Electroradiology, Jagiellonian University Medical College, Michałowskiego 12, 31-126 Krakow, Poland; pkarcz@su.krakow.pl; 4 Department of Child and Adolescent Psychiatry, Faculty of Medicine, Jagiellonian University, Medical College, Kopernika 21a, 31-501 Krakow, Poland; natalia.smierciak@uj.edu.pl (N.Ś.); marta.szwajca@uj.edu.pl (M.S.); maciej.pilecki@uj.edu.pl (M.P.); 5 Department of Clinical Genomics, Center for Individualized Medicine, Mayo Clinic, Rochester, MN 55905, USA; kozicz.tamas@mayo.edu

**Keywords:** schizophrenia, oxidative stress, brain metabolites, magnetic resonance spectroscopy

## Abstract

Schizophrenia is a complex mental disorder whose course varies with periods of deterioration and symptomatic improvement without diagnosis and treatment specific for the disease. So far, it has not been possible to clearly define what kinds of functional and structural changes are responsible for the onset or recurrence of acute psychotic decompensation in the course of schizophrenia, and to what extent personality disorders may precede the appearance of the appropriate symptoms. The work combines magnetic resonance spectroscopy imaging with clinical evaluation and laboratory tests to determine the likely pathway of schizophrenia development by identifying peripheral cerebral biomarkers compared to personality disorders. The relationship between the level of metabolites in the brain, the clinical status of patients according to International Statistical Classification of Diseases and Related Health Problems, 10th Revision ICD-10, duration of untreated psychosis (DUP), and biochemical indices related to redox balance (malondialdehyde), the efficiency of antioxidant systems (FRAP), and bioenergetic metabolism of mitochondria, were investigated. There was a reduction in the level of brain *N*-acetyl-aspartate and glutamate in the anterior cingulate gyrus of patients with schisophrenia compared to the other groups that seems more to reflect a biological etiopathological factor of psychosis. Decreased activity of brain metabolites correlated with increased peripheral oxidative stress (increased malondialdehyde MDA) associated with decreased efficiency of antioxidant systems (FRAP) and the breakdown of clinical symptoms in patients with schizophrenia in the course of psychotic decompensation compared to other groups. The period of untreated psychosis correlated negatively with glucose value in the brain of people with schizophrenia, and positively with choline level. The demonstrated differences between two psychiatric units, such as schizophrenia and personality disorders in relation to healthy people, may be used to improve the diagnosis and prognosis of schizophrenia compared to other heterogenous psychopathology in the future. The collapse of clinical symptoms of patients with schizophrenia in the course of psychotic decompensation may be associated with the occurrence of specific schizotypes, the determination of which is possible by determining common relationships between changes in metabolic activity of particular brain structures and peripheral parameters, which may be an important biological etiopathological factor of psychosis. Markers of peripheral redox imbalance associated with disturbed bioenergy metabolism in the brain may provide specific biological factors of psychosis however, they need to be confirmed in further studies.

## 1. Introduction

Schizophrenia is a chronic, debilitating neurodegenerative disease, which is characterized by, among others, behavioral changes and the occurrence of productive symptoms, presenting a wide spectrum of clinical syndromes, significant inter-individual differences, and biological heterogeneity at the level of analyzed patient groups [1,2]. Emerging heterogeneity of schizophrenia involving a number of neurochemical [3], genetic [4], metabolic [5], immunological, or biochemical changes [6], decides on still emerging problems regarding the diagnosis and differentiation of schizophrenia.

So far, it has not been possible to clearly define what types of changes of a functional and structural nature are responsible for the occurrence or recurrence of acute psychotic decompensation in the course of schizophrenia, which was the subject of our interest.

It is known from the literature that in the course of schizophrenia there are periods of deterioration and symptomatic improvement, accompanied by abnormal structural connectivity of the brain [7]. However, the answer to the question about the onset and course of schizophrenia and the participation of certain specific biomarkers (both cerebral and peripheral) of the disease has not been resolved so far [8]. According to catamnestic data, more than 70% of patients who develop acute symptoms of psychotic decompensation in the course of schizophrenia for the first time experience significant symptomatic improvement. However, acute symptoms reappear in most of them over time, despite the treatment, requiring hospitalization or a significant modification of the remission maintenance dose. Among patients who experienced the first episode of schizophrenia, various degrees of remission should be dealt with, from complete through partial to conditions in which no type of treatment will lead to symptomatic improvement. The negative symptoms of schizophrenia associated with withdrawal from life activities, difficulty in building and maintaining interpersonal relationships and changes in experiencing emotions are particularly difficult to treat. Productive symptoms, such as hallucinations characteristic for schizophrenia, formal disorganization of thinking or disturbance of its content in the form of delusions of exposure and influence, persecution, respond to an antipsychotic treatment to a greater extent.

Personality disorders treated differently by different classification systems (according to Diagnostic and Statistical Manual of Mental Disorders, fourth edition DSM-IV, a separate nosological unit in psychiatry, as opposed to International Statistical Classification of Diseases and Related Health Problems, 10th Revision ICD-10, which classifies them together with schizophrenia), are considered one of the risk factors or precede the development of schizophrenia. Treatment of schizophrenia is mainly based on the use of antipsychotic drugs [9]. In personality disorders, antipsychotics are used less frequently and in low doses [10]. The matter is even more difficult when it comes to the interpretation problems of these two systems, as it is postulated that common causative factors are involved in both schizophrenia and personality disorders. These are: Genetic predispositions, environmental factors [11], perinatal complications, the course of pregnancy [12], early childhood trauma [13], circadian rhythms, treatment with neuroleptics, intestinal dysbiosis, early childhood temperament (personality), advanced age of the father (>34 years) [14] with an increased number of de novo mutations in reproductive cells, the intelligence quotient (IQ) [15], lifestyle, factors that modify the natural (not modified by treatment) course of the disease, including the development of schizophrenia [16]. It has been mentioned that schizophrenia is characterized by a disturbance in brain development occurring in the very early stages of life, as was presented in our previous work, and that personality disorders are not neurodevelopmental disorders. Imaging studies show essential differences in patients with schizophrenia but not in healthy people or those with personality disorders. Such differences concern brain changes such as enlarged brain ventricles [17]: There is a 3% loss in brain volume compared to healthy individuals [18]. The reduction of brain volume applies in particular to certain structures: Amygdala, hippocampus, and thalamus [17,19,20,21,22].

There are differences in sleep disorders between people with personality disorders and those with schizophrenia. The most visible sleep abnormalities that occur in schizophrenia patients are increased sleep latency [23], reduced total sleep time [24], decreased sleep efficiency [25], increased total standby time [26], and shortened latency of rapid eye movement [27]. Insomnia is a common symptom of schizophrenia, regardless of treatment status (previously untreated or previously treated) or phase of the clinical course (acute or chronic). Insomnia in schizophrenia patients may in part be related to an overactive dopaminergic system. The GABAergic system may also be involved in sleep disorders in schizophrenia [28]. Benca (1992) proved in meta-analysis that sleep disorders are more common in people with schizophrenia compared to healthy individuals [29]. Another meta-analysis [30] showed that sleep disturbances were consistently present in untreated schizophrenia patients compared to healthy subjects. These results confirm that patients with schizophrenia have sleep disorders that are not necessarily a consequence of pharmacological treatment, suggesting that sleep disorders are an inherent feature of schizophrenia.

Sleep disturbances are not common in subjects with personality disorders [29]. It has been shown that patients with disturbed circadian rhythms more often suffer from personality disorders than healthy people [31]. In subjects with personality disorders the mechanisms causing sleep disorders are not the result of biological processes to such a high degree as in subjects with schizophrenia, and patients with personality disorders more often report a reduction in the subjective quality of sleep [32]. 

Undiagnosed personality disorders remain a significant problem in clinical practice, and ultimately lead to the ineffectiveness of the therapy [33]. The simultaneous occurrence of other mental disorders is very common, and the presence of personality disorders often has a negative impact on the further course of the disease. An additional problem for undiagnosed personality disorders is frequent suicides and high premature mortality, which more and more often necessitate a reliable diagnosis [34]. The heterogeneous nature of the disease determines the occurrence of phenotypically variable forms of the disease (described in the literature as the so-called schizotypes) [35] along with a clear deterioration of clinical symptoms, referred to in the literature as psychotic decompensation. The subject of the study was the changes in this period (psychotic decompensation), which concerned patients with schizophrenia who experienced a clear deterioration of their condition in terms of clinical symptoms. Due to the indications appearing in the literature of the heterogeneity of clinical symptoms of the classified groups of patients and the possibility of determining of the schizotypes resulting from different causative factors (a diagnostic approach based on causative factors facilitating the classification) [36], we decided to link the observed changes of marked clinical deterioration with the areas of individual brain structures in terms of cellular metabolism with simultaneous changes in peripheral markers in both analyzed groups. In the aspect of the multifactorial nature of schizophrenia, it seems important to link the above-mentioned factors that may also coexist among healthy people or people with personality disorders with central changes [37].

In the study, psychotic decompensation concerned patients with schizophrenia who experienced deterioration of the condition of patients with schizophrenia in terms of symptoms (the results of the overall positive and negative syndrome scale PANSS scale of all patients indicated a significant intensification of psychosis symptoms). The approach presented above will allow the creation of metabolic profiles characteristic of individual phases of the disease and, consequently, may contribute to facilitating the tracking of neurochemical changes and simplifying the process of making a diagnosis or selecting pharmacotherapy.

It is known from the literature that brain metabolites are used as valuable predictors differentiating schizophrenia from bipolar disorder. In the study by Coppens et al., nuclear magnetic resonance spectroscopy (MRS) was used to search for specific central biomarkers differentiating these two diseases [38]. The metabolites typical for schizophrenia were gamma-aminobutyric acid (GABA), isovaleryl carnitine, mannitol, and pantothenate, while in patients with bipolar disorder they were 2,3-diphospho-d-glyceric acid, monoethylmalonate, and *N*-acetyl aspartylglutamic acid [39]. Additionally, thanks to the NMR technique, changes in the concentration of these brain metabolites were found. *N*-acetyl-aspartate (NAA) in different regions of the brain turned out to be highly differentiating between these two diseases. In the case of schizophrenia, it was the thalamus and frontal lobe, and in the case of bipolar disorder, the basal ganglia.

Some borderline personality traits can resemble schizophrenia. By making a differential assessment based solely on clinical symptoms, one may overlook comorbidities associated with personality disorders that, if left untreated, may develop the underlying disease in the future [40]. Additionally, one of the important aspects in the treatment of schizophrenia is the phenomenological approach that treats personality disorders as the key to understanding the psychopathology of the schizophrenic process and defining the so-called schizotypes and disease phenotypes [41] resulting from different causative factors (a diagnostic approach based on causative factors facilitating the classification), e.g., environmental factors, experiences of pleasure, sleep disorders, early childhood trauma, which together or separately determine the further course of the disease [42,43].

In the differential diagnosis of schizophrenia and personality disorders, biochemical indices related to redox balance have been proved to be useful [44]. In order to differentiate schizophrenia from personality disorders, the genetic variability of one of the antioxidant systems was used as an indicator of the breakdown of the defensive barrier in response to oxidative stress. This system concerned, inter alia, methionine sulfoxide reductase system (MSRA), whose genetic dysregulation may be the cause of increased psychosis different from the physiological state related to mitochondrial dysfunction [45]. The role of mitochondria has been gaining importance recently, because in addition to producing ATP, mitochondria are also a significant source of reactive oxygen species, which, having an unpaired number of electrons, become extremely reactive and therefore toxic for cell structures, contributing to the activation of transcription factors sensitive to redox imbalance, acting as signaling molecules for the regulation of gene expression or participating in the activation of pro-inflammatory factors or the complement system [46,47]. In the context of schizophrenia, the function of mitochondria seems to be all the more crucial as it is directly related to the factors inducing the formation of changes in the brain already in the prenatal period. As it turns out, intrauterine hypoxia was the direct cause of increased biogenesis and mitochondrial content, which resulted in deregulation of mitochondria and decreased energy metabolism in brain astrocytes [43]. These changes also led to a decreased antioxidant defense and decreased expression of key genes of anaerobic metabolism in mice, which was a premise to verify this issue in our model.

In the statistical model we built, taking into account a number of variables, including neuronal, clinical, psychosocial, and biochemical factors related to redox balance, peripheral oxidative stress played an important role, which, in combination with central and clinical mechanisms, may be a valuable tool for assessing the course of the disease. We pay attention to the peripheral oxidative stress, which was significantly associated with central markers without being related to other peripheral parameters. The observed peripheral oxidative stress is secondary to neuronal abnormalities, but emphasizes the relationship with the probable phenotype of the development and progression of schizophrenia associated with redox balance and mitochondrial function as an important etiological factor of the course of psychosis [48]. This approach may facilitate the creation of a future model in which certain pathophysiologies of psychosis are associated with specific peripheral biomarkers associated with the course of schizophrenia.

Lack of a universal model taking into account predictors of the disease that are well confirmed in clinical trials and experimental studies indicates that only a multifaceted approach to the disease can ensure its effective therapy [49]. There are many hypotheses trying to explain the causes and mechanisms of this disease. Oxidant–antioxidant imbalance related to the function of the mitochondria discussed above [50], mitochondrial aberrations [43], loss of plasticity and reduction of neuronal mass [51], and insulin resistance [51].

Many brain mechanisms depend on energy production, i.e., ATP synthesis. Therefore, it is essential for the proper functioning of the nervous system to maintain the reliable state of mitochondria. One of the environmental factors, possibly damaging the mitochondrial function in the course of schizophrenia, is hypoxia. Research shows that exposure of astrocytes to hypoxia disrupts the parameters that reflect their functionality. There is a reduction in their lifespan along with the prolonged exposure. There is also a decrease in the cytosolic concentration of Ca^2+^ ions, while an intensification of ion uptake by mitochondria, which in turn may violate their membrane potential. Depolarization can alter the permeability of the membrane with subsequent disturbance in the concentrations of other key compounds. The accumulation of reactive oxygen species and the narrowing of the body’s antioxidant reserve are successively observed. Moreover, it was shown that during exposure to hypoxia, astrocytes produced lower amounts of ATP; accumulation of ADP, pyruvate, and lactate occurred, which suggests the dominance of the glycolytic pathway [43].

Both changes in the level of brain metabolites and biochemical parameters in the blood are already visible in the early stages of the disease and undergo changes during its duration [52,53]. In addition, the clinical condition and subsequent treatment outcome depends on the length of time that patients do not undergo antipsychotic treatment (DUP, duration of untreated psychosis) [54]. DUP is a phenomenon strictly limited to schizophrenia, sometimes also used in psychotic bipolar disorder. It is believed that in the case of schizophrenia there are two stages before the start of the treatment: Prodromal symptoms and sometimes untreated psychosis, when the symptoms of the disease are already increasing but the patient dissimulates them. Sometimes they are combined into one category. In many studies, DUP even after many years has an influence on, e.g., the course or results of psychosis treatment. The longer the period of untreated psychosis, the more serious the prognosis of therapeutic possibilities and the further course of the disease. DUP is considered as the period of progression of biological changes underlying schizophrenia. This progression can be inhibited by the initiation of treatment, hence the delay in its initiation is even harmful.

In the case of a group of patients with personality problems, it is difficult to unequivocally construct a measure analogous to that of DUP in schizophrenia. The course of personality problems is often inscribed in life and relational crises related to maturing, moving to the next stages of education, entering into relationships that are subject to intensification and dissolution. Not all patients experience behaviors such as self-harm or suicide attempts that are clearly defined when the symptoms start. It should also be noted that in the theory of personality development, its basic framework is shaped in the first years of life. Hence the moment of symptomatic decompensation is closely related not to a fundamental internal change, but to the appearance of such external or life circumstances that cause symptoms. 

The authors based on selected markers of anatomical and functional changes in the brain, which indicate significant modifications in the course of schizophrenia, including prefrontal, anterior, and posterior cortex, part of the cingulate gyrus, or thalamus [55,56], that are responsible for identifying and processing information, motivation, and motor coordination. These traits are impaired in the course of schizophrenia and translate into the appearance of positive symptoms such as hallucinations, delusions, disorganization of thoughts and statements, and coordination disorders, that determine the clinical diagnosis [57,58]. Using imaging techniques, i.e., magnetic resonance spectroscopy (MRS), allowed to determine the level of metabolites, such as N-acetyl-aspartate (NAA), glutamate (Gln), glucose (Glu), or choline (Cho), in the above-mentioned brain areas. These metabolites play a key role in neurotransmission, vitality and integrity of neurons, i.e., the activities responsible for conduction of nerve impulses that affect cognitive function [59,60]. 

Due to the heterogeneity of clinical symptoms of schizophrenic patients who predispose to the development of psychosis, in the presented study we attempted to determine the likely path of disease development by establishing common relationships between the levels of cerebral peripheral markers, which are a measurable effect of the deterioration in functioning expressed by the severity of clinical symptoms (psychotic decompensation).

Finding connections between changes in clinical symptoms associated with patients marked quality of life deterioration, in regard metabolite ratio alterations in particular brain areas and in regard to simultaneous changes in peripheral markers in the analyzed groups, may significantly improve the etiopathological nature of psychosis.

## 2. Materials and Methods

### 2.1. Participants of the Study

The study involved: 40 young patients (18 women and 22 men; average age 22.68 ± 7.39 years) diagnosed with paranoid schizophrenia (F20) during psychotic decompensation according to ICD-10 classification 41 young patients (35 women and 6 men; average age 22.24 ± 8.43 years) diagnosed with personality disorders (F60) according to ICD-10 classification; and 30 healthy volunteers (17 women and 13 men; average age 29.5 ± 5.33 years) without prior psychiatric diagnosis. Diagnosis was made by a psychiatrist based on a structured clinical interview, medical examination, and collected medical records. All patients were admitted to the Clinical Department of Adult Psychiatry and the Clinical Department of Child and Youth Psychiatry of the University Hospital in Krakow, Poland. The selected groups were characterized by relative uniformity of race, age, and body mass index.

The inclusion and exclusion criteria were contained in the study protocol. Each patient participating in the study gave informed consent. Patient data and diagnostic test results were stored and developed in anonymous way. Patients and their legal guardians were fully informed about the purpose and the course of the study.

Inclusion criteria were: Informed consent of the patient (in the case of teenagers under 17 consent was given by one of the parents), age between 15 and 40 years, requirement for psychiatric hospitalization, medical diagnosis of psychotic disorders (group with paranoid schizophrenia, F20) or personality disorders (F60).

Exclusion criteria were: age below 15 or over 40, lack of consent to participate in the study, partial legal capacity, foster care, mental disability of the parent in relation to minor patient, drug or psychoactive substances abuse, overuse of alcohol or other substances (other than tobacco), psychiatric diagnosis of affective disorders (F30–F39), use of the drugs (antibiotics, non-steroidal anti-inflammatory drugs, corticosteroids, vitamin preparations, antioxidants) in the last 2 weeks before the admission, the occurrence of chronic diseases, hospitalization without consent or from a court order. Patients that had contraindications for Magnetic Resonance Imaging (MRI) imaging, like strong claustrophobia and presence of metallic implants (such as cardiac pacemakers, cochlear implants), that have magnetic properties, were also excluded from the study.

The studied groups of patients with both schizophrenia and people diagnosed with personality disorders were carefully selected for the conducted research. In the case of people with schizophrenia in the course of psychotic decompensation, qualification concerned those who experienced deterioration of the condition in terms of symptoms (the results of the overall PANSS scale of all patients indicated a significant intensification of psychosis symptoms).

In schizophrenics who were the subject of our research (schizophrenia is a disease in which there is a number of structural and functional brain abnormalities, including gray matter, accompanied by the emergence of negative and productive symptoms resulting from them) at the moment of psychotic decompensation in the constructed model of a number of variables (clinical, neuronal, biochemical, psychosocial), the potential relationships of the biomarkers of the cerebral peripheral axis and the clinical state of patients were assessed.

Patients were selected for research in the period of psychotic decompensation due to the possibility of a relationship between central and peripheral mechanisms, which together may affect the occurrence of clinical symptoms severity.

The selection of the patients with personality disorders as additional to the conducted research is a significant reinforcement of this research project. The basis for inclusion in this group was the diagnosis made by a psychiatrist in accordance with the ICD-10 criteria during a medical examination, an interview, history of illness, and psychological diagnosis. Personality disorders are a group of very different states, from the psychotic cluster to internalizing and anxious features. Clusters B and C belong to the internalizing disorders, and A can be very chronic without ever changing to a full psychosis. The personality disorder group was a heterogenous group without a psychotic disorder and strong medication that belongs to that, while sharing some environmental etiological factors and stressors. The stress in personality disorders tends to be more chronic than deterioration of psychosis. 

An interesting question for us in the selection of this group was the question of the biological etiopathological factors of psychosis compared to other groups which are now treated differently by different classification systems. Namely, the American system of psychiatric classification DSM-IV, does not consider personality disorders in the same category as schizophrenia or depression, while the International Statistical Classification of Diseases and Related Diseases (ICD-10) Health Problems, 10th Revision) puts personality disorders on a par with other diseases, including schizophrenia.

Matched healthy subjects without a psychiatric history or prior use of narcotic psychoactive substances were recruited among health care professionals as a potentially healthy control group.

After the initial clinical evaluation, all study participants meeting the inclusion criteria were asked to participate in neuroimaging studies, i.e., head magnetic resonance imaging in combination with head magnetic resonance spectroscopy with 1.5 T scanner. The examination was performed in the Department of Diagnostic Imaging of University Hospital in Krakow.

Pharmacotherapy was prescribed and continued in all patients of psychosis group in accordance with the American Psychiatric Association guidelines for the treatment of acute psychosis and personality disorders [61,62,63]. Classic and atypical neuroleptics were used as monotherapy, as well as polytherapy at therapeutic doses not exceeding the recommended. No side effects of pharmacotherapy were observed in any of the patients. The study assessed the duration of untreated psychosis (DUP), which was estimated on the basis of historical data, i.e., the time elapsed from the first symptoms of psychosis to the implementation of pharmacotherapy [64].

Blood samples were collected during routine collection of material in the first week after the admission of the participants, as well as after 12 weeks. Patients were recruited from January 2019 to January 2020.

Informed written consent was obtained from all participants prior to involvement in the study. The study protocol was approved by the Local Research Ethics Committee, Jagiellonian University and was in accordance with the principles of the Declaration of Helsinki II. The study protocol was approved by the Bioethics Committee of the Jagiellonian University (consent number 1072.6120.152.2019 of 27 June 2019).

### 2.2. Clinical Evaluation

A detailed psychiatric and demographic evaluation including age, sex, and duration of untreated psychosis (DUP, i.e., a measure used in our studies only for psychosis) was performed in all patients during the admission. The severity of psychotic symptoms was assessed using the PANSS (Positive and Negative Syndrome) scale [47], which includes the following subscales: Positive symptoms (P1 to P7), negative symptoms (N1 to N7), general psychopathology (G1 to G16), and overall result (T). Positive symptoms (P) include: Formal thinking disorders, delusions, hallucinations, excessive agitation, grandiosity, suspiciousness, and hostility. Negative symptoms (N) include: Shortness of affect, emotional withdrawal, poor communication, passivity, abstract and stereotypical thinking disorders, and lack of spontaneity and fluency in conversation. G scale describes symptoms, such as: Concern for physical health, anxiety, tension, guilt, mannerisms and strange attitudes, depressiveness, motor inhibition, lack of cooperation, unusual thinking content, confusion, attention disorder, lack of criticism and insight, disorder of the will, and impulsive actions [65]. The assessment of the clinical state of the F60 group was based on psychiatric and psychological diagnosis.

### 2.3. Laboratory Tests and Therapeutic Treatment

After qualifying the participants for the study, 10 mL venous blood was collected to a closed Sarstedt system. Patients were fasting and collection took place at 7–9 a.m. Samples were stored at 4 °C and transported on ice. Preparation of the material for testing began no more than 4 h after collection. Samples with visible bilirubinemia, hemolysis, lipemia, and turbidity were discarded. Routine blood laboratory tests (performed in serum) included morphology, lipid profile: Triglycerides (TG), total cholesterol (TC), high-density lipoprotein (HDL), low-density lipoprotein (LDL), C-reactive protein (CRP), and ionogram (K^+^, Na^+^, Mg^+^), glucose. Laboratory tests were performed on the day of blood collection at the Hospital Clinical Laboratory in Krakow using a Sysmex XN-2000 automated analyzer (Cobe, Japan) for testing blood counts and Cobas 6000 and Cobas 8000 biochemical analyzers (Roche Diagnostics, Mannheim, Germany) for the assessment of biochemical and hormonal parameters. Accurate demographic and clinical data were collected, including duration and severity of psychotic symptoms prior to admission to the hospital, accompanying somatic diseases, previously used pharmacotherapy, as well as data on treatment with neuroleptics and complications during a hospital stay.

### 2.4. Assessment of Oxidant–Antioxidant Status

#### 2.4.1. Assessment of the Antioxidative Potential Expressed as FRAP

Total plasma antioxidant capacity, expressed as FRAP, is the result of the action of low molecular weight antioxidants (such as α-tocopherol, ascorbic acid, β-carotene, glutathione, uric acid, bilirubin), proteins (ceruloplasmin, ferritin, albumin, transferrin), and enzyme systems. FRAP reflects oxidative and antioxidant status, which shifts may be associated with increased oxidative stress and/or exhaustion of antioxidative defense mechanisms.

Total antioxidant capacity was assessed by measuring FRAP in peripheral blood in all study participants. Assays were performed on pre-prepared blood plasma (after separation from blood, plasma was collected and centrifuged for 15 min at 14,000× *g* at 4 °C and then stored at −80 °C for the analysis).

Total antioxidative potential of blood plasma for the reduction of Fe^3+^ ions was determined using the Benzie and Strain method [66]. The reduction of Fe^3+^ to Fe^2+^ takes place in the presence of tripyridyltriazine (TPTZ), which causes the formation of the Fe^2+^-TPTZ complex of an intense blue color. The experiment used plasma together with the following reagents from Sigma-Aldrich, St Louis, Mo, USA: 100 mL 0.3 M acetate buffer (pH 3.6), 10 mL 0.01 M TPTZ dissolved in 10 ml 0.04 M HCl solution at 70 °C and 10 mL 0.02 M FeCl_3_ · 6H_2_O. 0.001 M iron (II) sulfate was dissolved to the 0.1–1.0 mM concentrations and adopted as a standard. The working solution was prepared on the day of the analysis and contained: 100 mL acetate buffer, 10 mL TPTZ solution, and 10 ml ferric (III) chloride. A micromethod was used using 96-well microtiter plates. The reaction mixture contained: 15 µL tested plasma, blank with distilled water or standard in appropriate concentrations. Subsequently, 300 µL of working substrate solution was added and the contents were mixed. Absorbance was measured after 10 min incubation at 37 °C at a wavelength of *λmax* = 593 nm on a FLUOstar Omega spectrophotometer (BMG Labtech, Ortenberg, Germany).

#### 2.4.2. Evaluation of the Severity of Oxidative Stress Based on the Malondialdehyde Test

The method is based on the reaction of malondialdehyde with thiobarbituric acid (TBA), resulting in the final pink product. Fluorimetric determination according to the Aust’s method was modified to increase specificity [67,68]. The modification consisted in the use of MDA-TBA extraction with butanol and measurement of fluorescence in the organic layer [69].

In the presented method of fluorimetric determination of the TBA-MDA adduct, one can observe unique maxima of fluorescence emission and excitation at *λmax* = 553 nm and *λmax* = 532 nm, respectively.

Blood plasma was used in this study. The following reagents from Sigma-Aldrich (USA) were used to carry out the reactions: 3.75% trichloroacetic acid (TCA), 0.025 M hydrochloric acid (HCl), 0.0925% thiobarbituric acid (TBA), and 0.03% butylhydroxytoluene (BHT). The working solution was obtained by dissolving the basic reagent (TBA/TCA/HCl) in water (on the day of the assay), thereby obtaining a mixture containing 3.5 mL TBA/TCA/HCl, 10.5 mL H_2_O, and 0.21 mL BHT. 1,1,3,3-tetramethoxypropane, which hydrolyses in an acidic environment in a stoichiometric ratio to TBA, was used as a standard. Hydrolysis was carried out in 0.05 M hydrochloric acid at a room temperature for 10 min, followed by preparation of standard 1,1,3,3-tetramethoxypropane solutions in the range of 10–50 μmol/L. The ratio of a test sample to working solution was 125 to 375 μL. The contents of the tubes were mixed for 10 s with a micro-shaker and then heated in a boiling water bath for 15 min. The tubes were then immediately cooled and left on ice for 10 min. They were then centrifuged for 10 min at 4000× *g* at 4 °C. Consequently, 200 μl of the organic-butanol layer was carefully transferred to the wells of a black 96-well plate (OptiPlate-96F Black, Perkin Elmer, Waltham, MA, USA). Fluorimetric measurements were performed at an excitation wavelength (Ex) of 536 nm and an emission wavelength (Em) of 549 nm. Readings of results were taken with an Omega FLUOstar spectrophotometer (BMG Labtech, Ortenberg, Germany) after 10 min.

### 2.5. Validation of Biochemical Methods

To confirm the usefulness of MDA and FRAP determination methods in plasma, validation was carried out in accordance with European guidelines for validation of analytical methods [70]. To evaluate the calibration, eight standard scales were prepared and coefficients of variation were determined for each concentration. The differences in precision regarding extreme concentrations in the Snedecor F test were not significant. The established correlation coefficients (*r*) were between 0.99 and 0.95 and were higher than the assumed limit of ≥0.99. The correlation coefficient r differed significantly from zero. Linearity characterized the entire range of the calibration curve. Slope factors indicating method sensitivity were statistically significant. To assess absorbance, the fluorescence dispersion around the standard curve was determined along with the remaining standard deviations. The standard deviations of the Sm method, which show the calibration accuracy, were satisfactory and were equal to 0.0137 (for FRAP) and 0.0105 (for MDA). Calibration coefficients (1.14–5.54%) were satisfactory.

### 2.6. Neuroimaging Studies

Magnetic resonance imaging (MRI) was performed using the 1.5T (General Electric Healthcare, Milwaukee, WI, USA) magnetic field induction MR system with 8-channel head coil (receive only). The MR system was equipped with strong whole-body gradients ensuring an amplitude of 33 mT/m and a rise rate of 120 T/m/s on each axis, thus providing fast, accurate, and highly repeatable scans. Each participant was comfortably positioned supine in the head coil to minimize any motion during the scan. Each participant was monitored for adverse events during imaging. The examination was performed using a standard brain imaging protocol which includes the following sequences:

1. 2D T2 fast spin echo sequence in axial plane (5.0 mm slice thickness, 1.5 mm spacing, 3897 ms TR, 82 ms TE, 24 cm FOV, 320 × 256 matrix);
2. 2D T2 FRFSE-XL fast spin echo sequence in sagittal plane (5.0 mm slice thickness, 1.5 mm spacing, 4905 ms TR, 82 ms TE, 24 cm FOV, 320 × 256 matrix);
3. 2D T2 FRFSE-XL fast spin echo sequence in coronal plane (5.0 mm slice thickness, 1.5 mm spacing, 3819 ms TR, 83 ms TE, 24 cm FOV, 320 × 256 matrix);
4. 2D T2 FLAIR in axial plane (5.0 mm slice thickness, 1.5 mm spacing, 8000 ms TR, 144 ms TE, 2000 ms TI, 24 cm FOV, 256 × 192 matrix).

**Figure 1:**
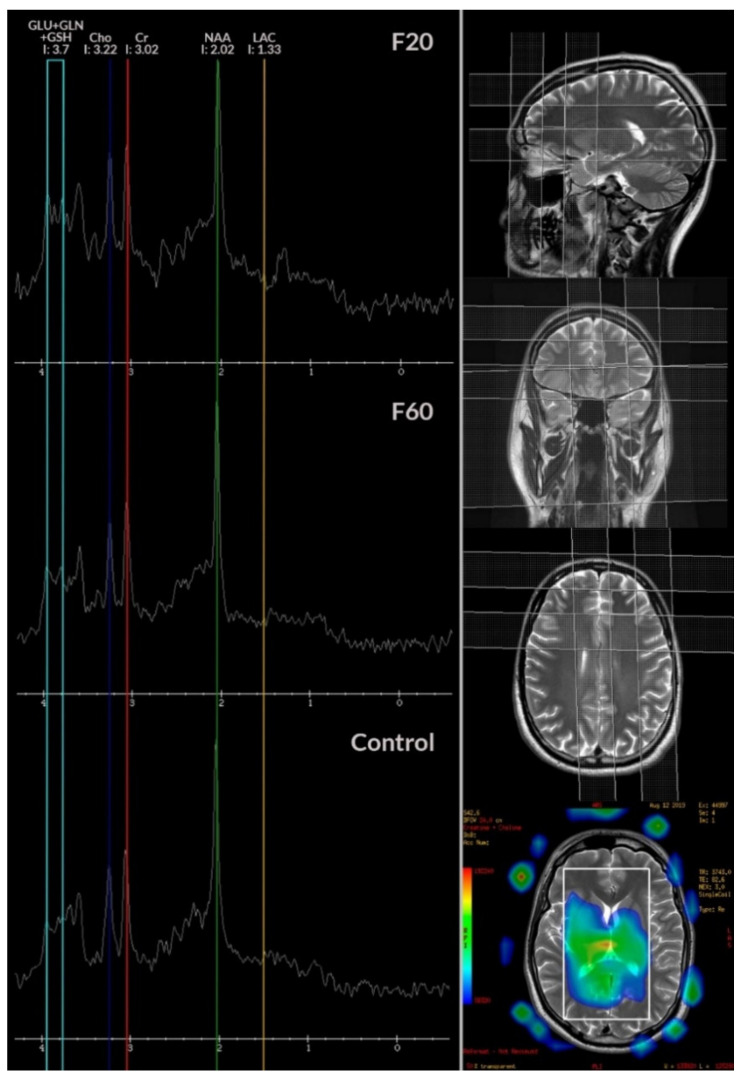
Magnetic resonance spectroscopy (MRS) maps showing regions of interest (ROI) and measurements in the anterior cingulate cortex (ACC) gyrus area of a patient with schizophrenia (F20) during psychotic decompensation (mean values of metabolite in this group significantly differed from personality disorders (F60) and control groups); Cho – choline; NAA – N-Acetylaspartate, LAC - lactate, Cr – creatine, Glu+Gln+GSH - glucose/glutamate/glutathione.

Two-dimensional T2-weighted images in axial, coronal, and sagittal plane were performed to visualize anatomical brain structures and plan position of the VOI (rectangle) for spectroscopy [71]. Spectroscopy was performed using a single-voxel technique (SVS). The MRS spectra were acquired using the point-resolved spectroscopy sequence (PRESS Point- Resolved Spectroscopy Sequence). PRESS sequence utilizes one 900 and two 1800 radiofrequency pulse. For water suppression, CHESS sequence (CHEmical shift Selective Imaging Sequence) was used with a frequency-selective 900 pulse to selectively excite the water signal, followed by a dephasing gradient. To obtain good quality spectra, automatic shimming was used. The magnetic resonance spectroscopy (MRS) acquisition parameters were: 35 ms TE, 1500 ms TR, 64 averages were acquired. In this study, MRS signal was collected from three locations distributed symmetrically in the left and right frontal lobes and the anterior cingulate cortex (ACC), parallel and superior to the dorsal anterior surface of the corpus callosum, and centered on the interhemispheric fissure. The volume of interest (VOI) was approximately 8 cm^3^. The size of VOI was adjusted to the anatomical size of the area the spectrum was collected from. The duration of the sequence was 2 min and 12 s. MRS data were analyzed using SAGE 7.0 (Spectroscopy Analysis by GE). The following metabolites were manually selected from the spectrum: lipids (lip 0.9–1.0 ppm), lactates (lac 1.33 ppm), alanine (ala 1.48 mm), *N*-acetyl-aspartate (NAA 2.02 ppm), glutamate (glu 2.1 and 3.7 ppm), γ-aminobutyric acid (GABA 2.3 ppm), glutamine (gln 2.45 and 3.7 ppm), creatine (Cr 3.02 and 3.9 ppm), choline (Cho 3.22 ppm), glucose (glc 3.43 and 3.8 ppm), myo-inositol (mI 3.56 ppm), and glutathione (GSH 3.7 ppm). A qualitative (visual assessment) and quantitative analysis of the reconstructed MRS spectra was performed (Figure 1). The concentration ratios of each metabolite were then determined relative to the total concentration of creatine (e.g., NAA/Cr). All the values were calculated for each VOI.

### 2.7. Statistical Analysis

Statistical analysis was performed using the IBM SPSS Statistics 25 package, New York, USA. Kruskal–Wallis test allowed to check the presence of any statistically significant differences between the groups. In case of statistically significant differences, an appropriate post-hoc test was used. The selection was based on the homogeneity of variances in the compared groups. *U*-Mann–Whitney test was used for the comparison of two groups. Spearman’s correlation analysis allowed to check whether there is a statistically significant relationship between the studied variables. Regression analysis had allowed to build a statistically significant model that had the greatest impact on the quality of life of the respondents. A statistically significant level was set to *p* < 0.05.

## 3. Results

### 3.1. Participants of the Study

This study was conducted on 111 young adults aged 22–29 years. 40 patients (18 women; with mean age ± standard deviation: 22.68 ± 7.39 and 22 men: 22 ± 55) with diagnoses of paranoid schizophrenia in the course of psychotic decompensation according to the ICD-10 classification (F20); 41 patients (both women and men with mean age and standard deviation; 22.24 ± 8.43) with diagnoses of personality disorders according to ICD-10 (F60) and matched with age 30 healthy control group were recruited to the study. 

Due to the fact that there were no statistically significant differences in terms of gender and age for the studied parameters (*p* > 0.05), subsequent analyses were performed without division into groups of females and males. Also, no statistically significant difference was detected between the studied groups regarding number of hospitalization, and duration of schizophrenia by years and number of episode.

### 3.2. Clinical Evaluation

Clinical symptoms and patients’ quality of life were evaluated using the Positive and Negative Syndrome Scale (PANSS) which describes the severity of positive (Positive scale, P), negative symptoms (Negative scale, N) and general psychopathology (General Psychopathology scale, G) and the overall score (PANSS Total, T). Schizophrenic patients assessed in the first time period (at admission to the ward) with P scale showed mean results ± standard deviation: 27.95 ± 6.61. According to N scale mean results ± standard deviation: 25.5 ± 5.57. General psychopathology exhibited mean results ± standard deviation: 53.98±10.45. While the mean ± standard deviation for total result was 106.33 ± 20.53; *p* < 0.001, Wilcoxon test.

Table 1 presents descriptive statistics on the history of the disease, including the age of the occurrence of psychosis and the time of untreated psychosis (DUP) of patients diagnosed with paranoid schizophrenia during psychotic decompensation.

### 3.3. Laboratory Analysis

Assessment of blood morphology and biochemical parameters in the three examined groups showed the existence of statistically significant differences between the levels of sodium, glucose, cholesterol, and high-density lipoproteins (HDL). Post-hoc analysis indicated that the average sodium level in the control group (mean ± standard deviation (SD): 138.73 ± 1.74) was significantly lower compared to both examined groups F20 (140.05 ± 1.93; mean ± standard deviation; *p* < 0.001) and F60 (141.35 ± 1.85; mean ± SD; *p* < 0.001) group. Statistically significant differences were observed between patients with schizophrenia in the course of psychotic decompensation (F20) and patients diagnosed with personality disorders (F60) (*p* = 0.03). In turn, the average glucose level in the F60 group (4.43 ± 0.52, mean ± SD) turned out to be significantly lower compared to the F20 group (5.09 ± 1.08, mean ± SD), and the control group (5.25 ± 0.9, mean ± SD; *p* < 0.001). The average cholesterol concentration in the F60 group was significantly lower compared to the control (*p* = 0.049) and F20 group (*p* = 0.048). However, the average HDL concentration in the schizophrenia patients turned out to be significantly lower compared to the control group (*p* = 0.02).

In the case of cortisol and complement C3 concentrations, group with schizophrenia had significantly higher levels compared to personality disorder’s patients.

Based on the calculations made for laboratory parameters (blood count and biochemical tests) that showed statistically significant changes in the examined groups, a quantitative assessment of the strength of the obtained test results was made by determining the size of the effect (eta-square, *eta^2^*): sodium 0.23 (23%, test power = 99.7%), glucose 0.12 (12%, test power = 86%), cholesterol 0.08 (8%, test power = 66.4%), HDL 0.09 (9%, test power = 77.3%), cortisol 0.39 (39%, power test = 100%), complement C3 0.49 (49%, test power = 100%). Sodium was the marker that distinguished three groups most.

### 3.4. Oxidant and Antioxidant Status Assessment

Statistically significant differences were observed between the three compared groups in terms of FRAP and MDA parameters in the first time period. The conducted pairwise comparisons indicated that the average FRAP level was significantly lower in schizophrenia compared to the control group (*p* < 0.001) and personality disorders (*p* < 0.001). In the case of lipid peroxidation index (MDA), its average level in the F20 group turned out to be significantly higher compared to the control group (*p* < 0.001) and F60 group (*p* < 0.001) (Table 2).

### 3.5. Neuroimaging Studies

Based on the magnetic resonance spectroscopy (MRS) study, it was evaluated, whether there were significant differences in the level of metabolites between the groups in terms of individual brain structures. There were no significant differences in terms of right and left frontal lobe parameters. In the case of anterior cyngulate cortex (ACC), significant differences concerned *N*-acetyl-aspartate (NAA 2.02) and glutamic acid (GLN 2.45). People with schizophrenia (F20) had a significantly lower mean (*p* = 0.049) NAA 2.02 compared to the F60 group. People from the F20 group had a significantly lower mean GLN 2.45 compared to the control (*p* = 0.001) and F60 (*p* = 0.003) groups (Table 3).

The *eta^2^* factor of effect size was equal to 0.04 (4%, test power = 34%) for *N*-acetyl-aspartate (NAA) 2.02, and 0.18 (18%, test power = 96.2%) for GLN 2.45.

In the case of assessment of the ratio of metabolites studied in the ACC area, significant differences concerned the glutamine (Gln)/creatine (Cr) ratio (GLN/CR). People with schizophrenia obtained a significantly lower mean value compared to the group with personality disorders (*p* = 0.01) and controls (*p* = 0.04) (Table 4). The *eta^2^* factor was equal to 0.12 (12%, test power = 80%).

### 3.6. Assessment of the Relationship between Brain Parameters and Clinical Condition

In the next step, the presence of a statistically significant relationship between the quality of life and brain parameters was evaluated. There were several significant relationships, out of which the strongest concerned GLU + GLN + GSH 3.7 for ACC with a positive (P) symptom scale. The higher the value of this ratio, the higher the score of the P scale (Table S1).

In the case of the ratios of the variables, there were individual statistically significant relationships. The strongest of them concerned GLC/CR for ACC with the scale of negative symptoms (N). The higher the value of this ratio, the lower the score of the N scale (Table S2).

When assessing the relationship between brain parameters and patients’ condition regarding the history of illness in the F20 group, the following statistically significant relationships are noteworthy. The number days of hospitalization correlates with the level of glucose in the left frontal lobe GLC 3.8 (ρ = –0.42; *p* = 0.02) in a significant way. In addition, hospitalization length correlates significantly with the GLU/CR ratio in the ACC area (ρ = –0.45; *p* = 0.02). In addition, it was observed that the age of the first episode correlated in a significant way with the level of glutamic acid (GLN 2.45) in the left frontal lobe area (ρ = –0.47; *p* = 0.007). Statistical parameters for a ratio of glutamic acid and creatine in the left frontal lobe (GLN/CR) were: ρ = –0.41; *p* = 0.02. 

In the case of untreated DUP psychosis, statistically significant relationships related to glucose level (GLC 3.43) in the right frontal lobe (ρ = –0.36; *p* = 0.04). In addition, the higher the DUP value, the higher the choline (CHO 3.22) value in the ACC area (ρ = 0.38; *p* = 0.03).

**Figure 2:**
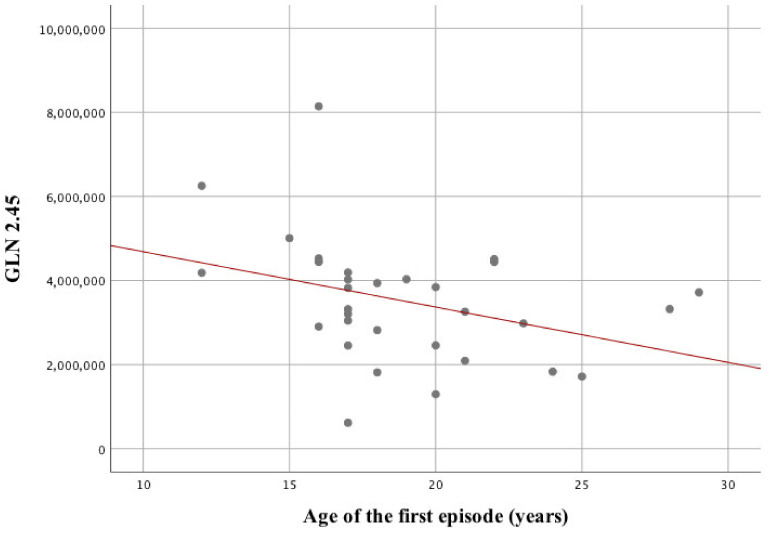
Relationship between age of the first episode (years) and GLN 2.45.

The strongest of the above relationships was between the age of the first episode and the value of GN 2.45 glutamic acid (Figure 2). No other statistically significant relationship was observed.

Consequently, a statistical model consisting of significant predictors which had the greatest impact on the quality of life (measured by the T scale) was created. Table 5 presents significant predictors included in the statistical model, beta value, and statistical significance. This model is statistically significant, F (7;24) = 5.9; *p* < 0.001.

GLN 2.45 glutamic acid in the area of the left frontal lobe turned out to be the strongest predictor of the quality of life. This model explains 53% of the variance of the T scale scoring.

### 3.7. Assessment of the Relationship between Oxidant–Antioxidant Balance Parameters, Routine Laboratory Markers, and Brain Metabolites

The relationship between the oxidant–antioxidant balance parameters, i.e., FRAP and MDA, and individual biochemical parameters, was assessed (Tables S3 and S4).

**Figure 3:**
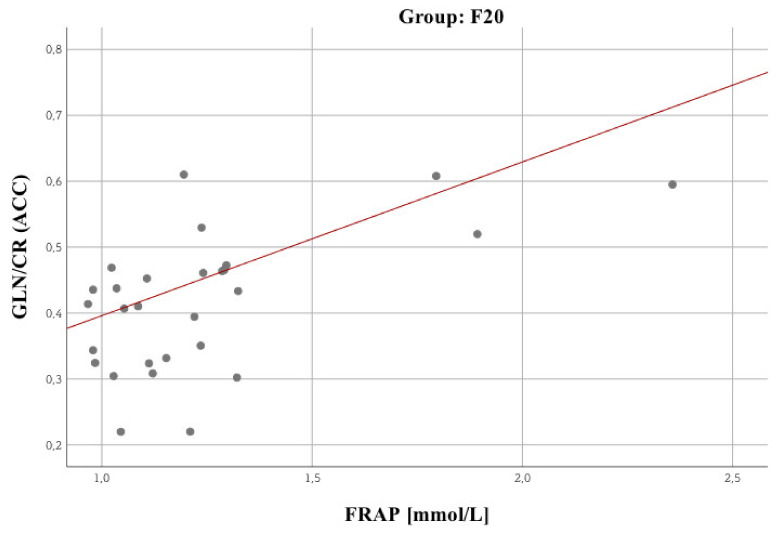
Correlation between FRAP and GLN/CR (ACC) in the group of people with schizophrenia (F20).

In terms of blood morphology and biochemical parameters, a small number of statistically significant relationships was observed. Two stronger relationships are noteworthy, namely between the chloride concentration in the F60 group (*R* = –0.42; *p* = 0.04, Spearman’s correlation). The reverse is true for MDA (*R* = 0.59; *p* = 0.003). Glucose showed statistically significant correlation with Total Antioxidant Potential (FRAP) in schizophrenia patients (F20) (*R* = 0.34; *p* = 0.03) and with malondialdehyde peroxidation product (MDA) in personality disorder patients (F60) (*R* = 0.43; *p* = 0.04, Spearman’s correlation). Cortisol showed statistically significant negative correlation with MDA in schizophrenia patients (F20) (*R* = –0.34; *p* = 0.04, Spearman’s correlation). As for the other biochemical parameters, there were other significant relationships. Attention was drawn to several of them, showing that: The higher the FRAP value, the lower the GLC 3.8 (*R* = –0.61; *p* = 0.002) value for ACC. The higher the FRAP value, the higher the GLN/CR (*R* = 0.62; *p* = 0.001) value for ACC (the strongest observed relationship, Figure 3).

**Figure 4:**
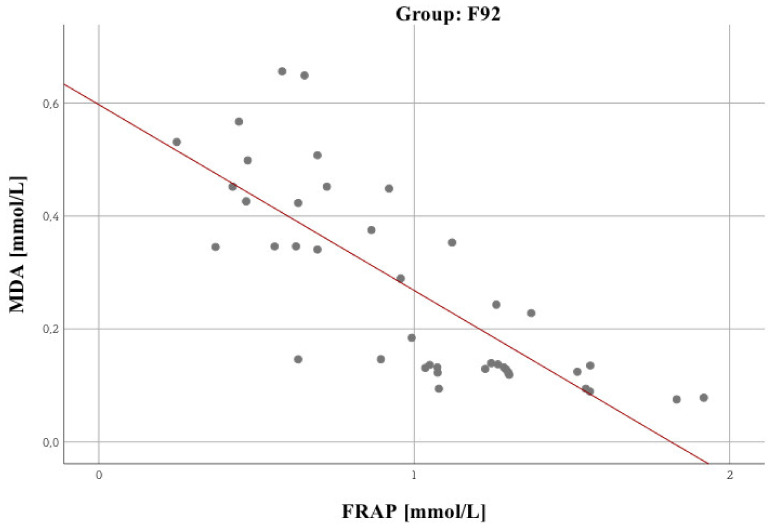
Correlation between FRAP and MDA in F60 group.

A very strong relationship was observed between FRAP and MDA in the F60 group (personality disorders): *r* = –0.82; *p* < 0.001 (Figure 4). No such regularity was observed in both remaining groups.

**Figure 5:**
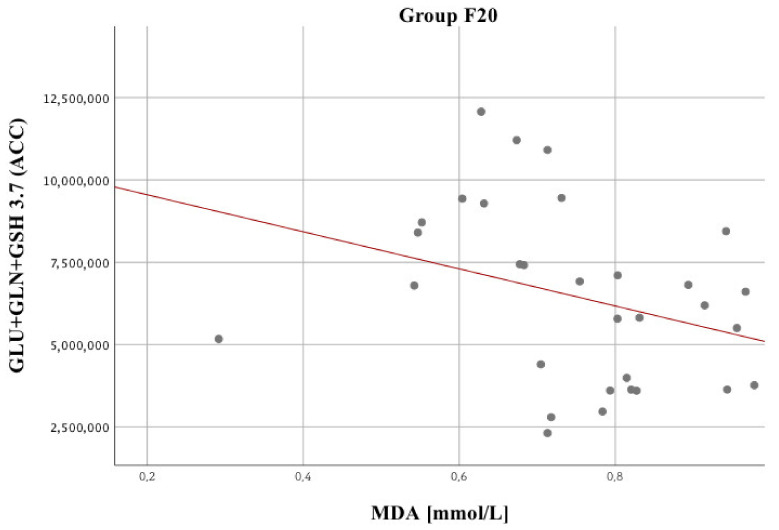
Significant correlation between MDA and brain metabolites, i.e., GLU + GLN + GSH 3.7 (glucose, glutamate, glutathione) in the ACC area.

**Figure 6:**
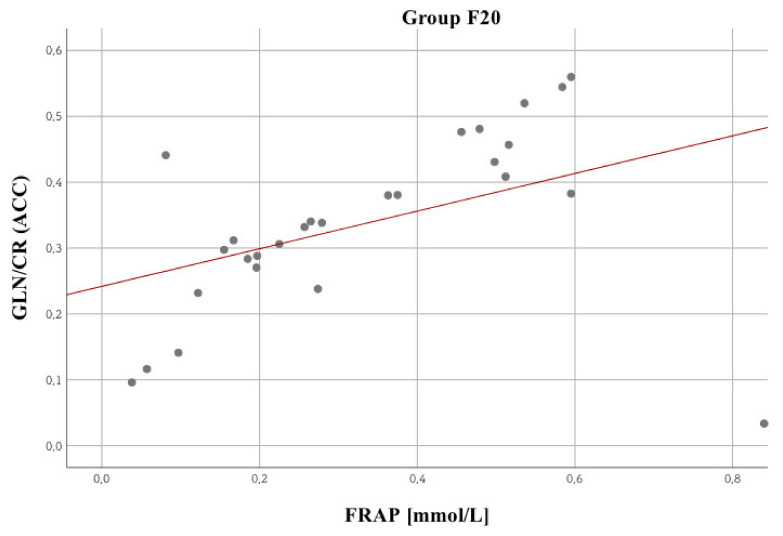
Significant correlation between the level of total antioxidant potential expressed as FRAP and brain metabolites, i.e., the ratio of glutamic acid to creatine (GLN/CR) in the ACC area.

Figure 5 shows a significant relationship between MDA and brain metabolites, i.e., GLU + GLN + GSH 3.7. Figure 6 presents a statistically significant GLN/CR vs FRAP correlation. No significant relationships were identified for NAA/CR.

## 4. Discussion

To the best of our knowledge, this study is the first to suggest severity-specific changes in the assessment of glutamate, *N*-acetyl-aspartate, and other ACC metabolites in the brain associated with the clinical status, untreated psychosis, and redox imbalance related to the severity of the psychotic process underlying psychotic decompensation in schizophrenia in comparison to personality disorders and healthy persons.

In the group of patients with schizophrenia, we recorded a typical ACC pattern in the corpus callosum region. According to our earlier hypothesis and limited data, the observed changes in glutamate levels can be associated with the mitochondrial metabolism of the brains of patients with schizophrenia. The role of mitochondrial function in the etiopathogenesis of schizophrenia has recently gained importance, as indicated by genetic, proteomic, enzymatic, and anatomical abnormalities [71,72]. An additional argument linking the appropriate patterns of brain metabolites with the changes in the brain’s bioenergy balance are the results indicating a reduction in the ability of pyramidal neurons to generate bioenergetic substrates from glucose via glycolytic pathways and the observed decrease in lactate production in schizophrenic patients. This is because neurons are unable to take up sufficient glucose for glycolysis, which entails intracellular depletion of the pool of available pyruvate/lactate for electron transport to the mitochondria, ultimately inducing energy deficits leading to TCA cycle abnormalities and impaired oxidative phosphorylation [73]. 

In addition to the key role in providing energy needed for the brain, mitochondria have been recognized as risk factors for mental disorders, including schizophrenia [74], bipolar disorder (BD) [71], and depression [75]. In the classical form, mitochondrial diseases are associated with the formation of complex I [76], although other mitochondrial abnormalities include oxidative damage [77], the increase in brain lactate levels as a result of abnormal glucose metabolism [78], or change in expression of mtDNA-encoded genes [79,80]. Impaired mitochondrial function due to large somatic mutations of mtDNA leads to their accumulation in brain tissue contributing to the increased production of reactive oxygen species (ROS) [80]. 

The research showing a reduction in the expression of genes related to oxidative phosphorylation in pyramidal neurons [81] confirms the theory of mitochondrial damage in schizophrenia. There are deficits in the genes for lactate dehydrogenase A, NADH dehydrogenases, and ATP synthase. These changes are specific for schizophrenia, but they were not found in MDD or bipolar disorder [82,83]. The reduction of mitochondrial function in combination with the incorrect use of energy substrates in neurons leads to a disturbance of metabolism and signaling of key proteins ensuring the maintenance of the proper formation of dendritic spines, the lower density (deficit) of which contributes to inhibiting the transmission of information received by neurons in schizophrenia [84].

Changed redox balance (associated with abnormal functioning of Na^+^/K^+^-ATP-ase or Ca^2+^ transport) and chronic inflammation of varying severity are visible in the studied group of patients with schizophrenia [85]. Bioenergetic abnormalities related to redox regulation in mitochondria are believed to be one of the major contributors to chronic schizophrenia [86], including deficits in energy storage and use in the brain. Redox homeostasis is deregulated after hypoxia [43] and in addition, in response to central changes, there is a compensatory breakdown of peripheral antioxidant systems [77]. Confirmation of this phenomenon is the disruption of bioenergy pathways in the dorsolateral prefrontal cortex (DLPFC) of patients with schizophrenia and control subjects in whom a decrease in hexokinase (HXK) and phosphofructokinase (PFK) activity in DLPFC was observed, as well as a decrease in PFK1 mRNA expression. This suggests that the risk of the genetic makeup of this disease is related to the essential role of bioenergy substrates [87] leading, inter alia, to impaired glucose utilization in these brain regions [88]. 

The mitochondrial theory regarding the development of schizophrenia is also confirmed by the microarray studies showing a significant reduction in the expression of genes encoding proteins related to the malate cycle, tricarboxylic acid (TCA), as well as ornithine-polyamine, aspartate-alanine and ubiquitin in the dorsolateral pregranular cortex (DLPFC) of patients with schizophrenia. This suggests impaired mechanisms of energy storage and use in the brains of patients with schizophrenia [89].

Magnetic resonance spectroscopy (MRS) studies provide direct evidence for the role of mitochondrial bioenergetic dysfunction as the primary etiological factor of schizophrenia [90]. In the studies of Du et al., the observed decrease in creatine kinase activity [91] confirms the decreasing trend of creatinine concentration in the ACC area in the brains of patients with schizophrenia, obtained in our research. This fact proves the essential role of enzymes in a critical state, which is psychotic decompensation in schizophrenia, in order to maintain a stable level of ATP during altered neuronal activity [92]. Other MRS studies on previously untreated schizophrenic patients suggest that reduced availability of high-energy phosphate may be a hallmark of the disease [93].

Oxidative stress caused by mitochondrial dysfunction may result in impaired defense mechanisms against free radicals (observed decrease in total antioxidant potential expressed as FRAP). The FRAP decrease obtained in this study reflects the body’s ability to counteract the effects of the oxidative stress metabolites not only in the brain, but also in the periphery. The presented results on the total antioxidant capacity decrease in patients in the course of psychotic decompensation prove the destabilization of this mechanism and explain the causes of increased lipid peroxidation (excessive malondialdehyde production). According to the mechanism proposed above, these disorders are directly associated with impairment of mitochondrial network dynamics, which may initiate various disorders of the nervous system, including inflammatory pathologies. The results obtained in this study are confirmed by the 2018 Sedlak et al. study, who observed the occurrence of peripheral antioxidative system dysregulation associated with a decrease in peripheral GSH as a possible pathological phenotype of the disease explaining the relationship between peripheral changes and oxidative stress in the brain [94]. Preclinical GSH deficiency described in the cited study (vs decrease in FRAP and increase in MDA in our work) correlated with the changes in the entire brain (and with the selected ACC region in our study) of patients with schizophrenia during psychotic decompensation. An additional confirmation of the existence of a strong network connecting oxidative stress in the brain with peripheral stress is a strong positive correlation between peripheral and brain GSH levels after sulforaphane treatment, which would explain the clinical phenotype of the disease among people with schizophrenia in our studies. Sulforaphane, crossing the blood–brain barrier, has dose-dependent effects on the brain. It induces the anti-inflammatory/antioxidant hemoxygenase-1 (HO-1) gene and protects against mitochondrial DNA mutations in different parts of the brain by improving negative symptoms and cognitive deficits in schizophrenia, stroke, and traumatic brain injury. The use of a combination of clinical phenotypes of the disease with peripheral biomarkers can provide information on the progression of these diseases and the effectiveness of pharmacological intervention [95]. 

The observed decrease in the level of metabolites in the ACC region (mainly Glu, as well as Gln, Cho, NAA) are associated with the synaptic follicle cycle, glutamate transmission, mitochondrial fusion, calcium buffering, and regulation of redox balance. This regulation concerns the production and removal of reactive oxygen, nitrogen or sulfur (ROS/RNS/RSS) depending on each other. These disorders decide on the observed profiles of changes in the level of brain metabolites in schizophrenia [96] and consequently the appearance of certain phenotypes of the disease [72]. Both the level of glutamate release measured by vGLUT and the number of glutamatergic synapses are varied in the level of brain-related anatomical areas of schizophrenia. Similar observations to those obtained in our studies concerning the decrease in glutamate and N-acetyl-aspartate levels in the area of anterior cingulate cortex (ACC) were made in the work of Reid et al., where the authors observed that glutamatergic metabolites and NAA may be reduced in the early stage of the disease [90]. On the other hand, elevated levels of these metabolites may suggest a biological etiopathological role of psychosis phase compared to personality disorders and control. This finding seems more to reflect a biological etiopathological role for further research aimed at examining the level of association of these metabolites with other factors, both psychosocial, environmental and peripheral markers, in order to clarify the pathomechanism of the disorders underlying cognitive deficits in schizophrenia. 

In this study, the area affected by the largest changes in the brain is the ACC area, where the decrease in synaptic density based on the reduced level of glutamate is the largest and may be associated with a lower number of cortical synaptic connections and thus, insufficient energy production in the form of ATP [74]. As a consequence of the observed changes, the presented data seems more to reflect a biological etiopathological role of psychosis mentioned in our earlier hypothesis that disruption of key mitochondrial functions may initiate the emergence of acute psychosis and, consequently, contribute to the development and modification of the course of schizophrenia [97]. 

In addition, the relationship between glutaminergic transmission of the brain connection network (in the ACC region), mitochondrial function, and oxidative stress on the periphery registered in this study is influenced by the duration of untreated psychosis (DUP) in schizophrenia during psychotic decompensation. The association between longer DUP and accelerated loss of specific brain structures depending on the time of pharmacological intervention suggests that psychosis may have a lasting, possibly detrimental effect on specific brain structures [98]. Strong positive relationship between DUP and the value of choline in the ACC area obtained in our research seems more to reflect a biological role like the results by Théberge et al. [99].

Despite many methodological differences, progressive reductions in NAA could be observed in various brain regions involved in the pathogenesis of schizophrenia [100]. As for the decreases in choline, creatine, and myoinositol levels obtained in this study in the course of psychotic decompensation, they are not reflected in other studies in which the levels of these metabolites largely remain unchanged in the stable phase of the disease, and the role of glutamate depending on the severity of schizophrenia could be an interesting etiopathological factor in further spectroscopic studies.

As the literature on the subject indicates, the structure and function of mitochondria largely depends on the use of both classic and atypical antipsychotic drugs [101]. Both classes of neuroleptics block the activity of complexes I and IV [102], which are the components of cytochrome C oxidase [103] and can be associated with the most commonly appearing dyskinesia (mainly first-generation neuroleptics), collapse of mitochondrial membrane potential, mitochondrial edema, severe lipid peroxidation (increase in MDA), and increase in oxygen, nitrogen or sulfuric free radicals, which ultimately induce apoptosis of dopaminergic neurons [104]. 

Haloperidol, the most commonly used classic neuroleptic, has a blocking effect on the sigma 1 receptor located on the mitochondria-associated endoplasmic reticulum membrane causing the accumulation of calcium [105] and iron [106]. According to the current knowledge, atypical neuroleptics (SGA) reduce the expression of genes encoding mitochondrial complexes as well as Mfn-2, Drp-1 genes encoding fusion and cleavable proteins. This effect was mainly observed in the treatment by olanzapine and clozapine [107]. The use of antipsychotic drugs also causes depolarization of mitochondrial membranes by affecting Ca^2+^, Zn^2+^, K^+^, and Cl^−^ channels [72], disruption of microvascular bioenergetics (HBVEC) in the blood–brain barrier [108], and a change in protein gene expression related to the process of glycolysis and oxidative phosphorylation [109]. The literature on the subject indicates, that during pharmacotherapy there is a disruption of electron transport in the respiratory chain, a decrease in the level of NADPH-Q reductase which results in a decrease in the production of ATP molecules [110]. The expression of antioxidative genes, i.e., SOD2, PKD1, and NAPG (classic neuroleptics), or BDH1, ACAMD, and NAGS (atypical neuroleptics), as well as the level of proinflammatory cytokines, is also reduced [111]. Campo et al. showed that the use of olanzapine causes a reduction in Acetyl-CoA levels in the mitochondrial cytosol and a weakening of insulin signaling by reducing the phosphorylation of Akt and reducing the expression of Opa-1 protein via the Akt-mTOR-NFκB-Opa-1 signaling pathway [109,112] as we wrote in our previous work [50].

**Figure 7:**
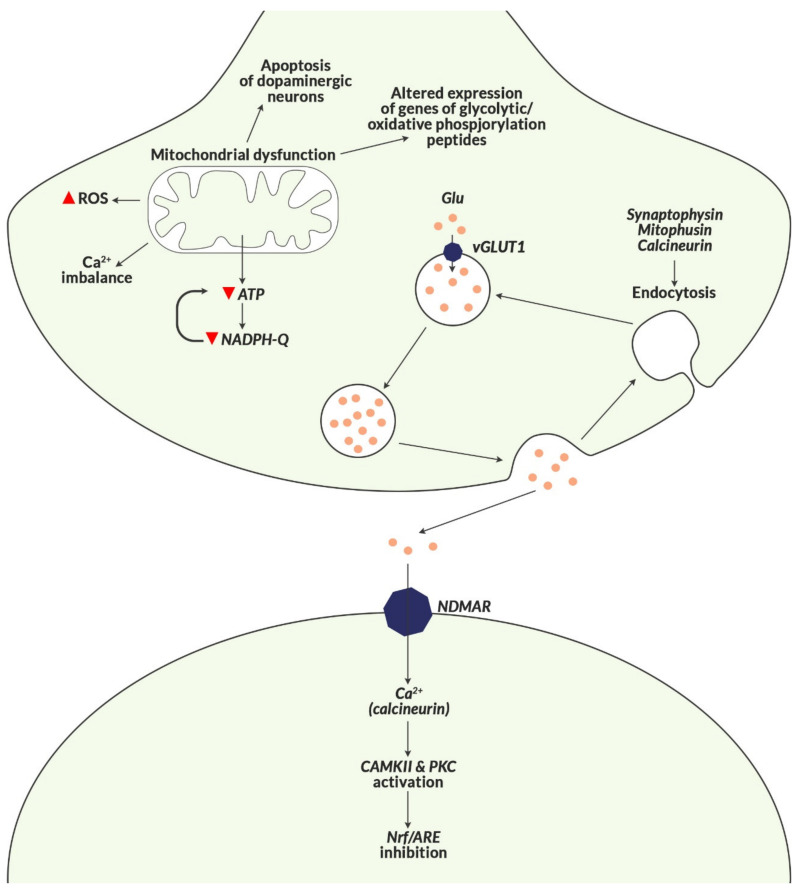
Axonal glutaminergic transport and its relationship with mitochondrial function. Vesicular glutamate transporters (vGLUT1) introduce glutamate into synaptic vesicles. The amount of vGLUT corresponds to the amount of glutamate release (reduced level of glutamate is associated with reduced synaptic release). It comes to a fusion of synaptic vesicles and endocytosis, which occurs thanks to synaptophysin (axon end marker) and mitofusin-2 found in mitochondria. Calcineurin, on the other hand, binds calcium with calmodulin in the brain by regulating the synaptic follicle cycle, which ensures constant circulation of an appropriate pool of synaptic vesicles and regulates endocytosis of synaptic vesicles during periods of intense synaptic activity. Postsynaptically, calcineurin activity is involved in the regulation of dopaminergic signal transduction dependent on the NMDA synaptic plasticity receptor. Glutamate activates NMDAR during depolarization and binding to their postsynaptic receptors, leading to Ca^2+^ entry into the postsynaptic neuron. As a consequence, Ca^2+^ and calmodulin-dependent protein kinase II (CaMKII) and protein kinase C (PKC) are activated. These, in turn, inhibit basic signaling pathways mediated by the Nrf/ARE transcription factor providing protection against oxidative stress [114].

The key amino acids produced with the participation of mitochondria, like glutamate (transformed into GABA in mitochondria), as well as the family of neurotransmitters, i.e., serotonin, noradrenaline, adrenaline and dopamine, are also of significance [113]. ATP produced in glycolysis in mitochondria is the driving force of these processes. Mitofusin-2 is a mitochondrial membrane protein that contributes to the maintenance and functioning of a normal mitochondrial network (Figure 7).

In the case of reactive oxygen species (ROS) such as superoxide anion radical (O^.-^_2_) produced in oxidative phosphorylation in mitochondria, reactive nitrogen species such as peroxynitrile (ONOO), and their mutual interactions, lipid, protein, and nucleic acid oxidation occurs. It can lead to toxic damage and death of nerve cells (neurodegeneration) in schizophrenia [72]. During these changes, ROS levels are dysregulated and when the observed increases in ROS are prolonged, which may result in matrix damage of mitochondria, that lose the ability to transfer electrons due to excess ROS/RNS generation [115]. This results in reduced electron flow in the electron transport chain, reduced ATP production, and consistently increasing oxidative stress-dependent accumulation of mitochondrial Ca2+ levels. Cellular metabolism is weakened and glycolysis is becoming a more preferred pathway for energy production resulting in accumulation of lactate in mitochondria and changes in their structure. Increased lactic acidosis as well as reduced extracellular pH are features characterizing damage to mitochondrial metabolism, which is also observed in our studies.

Chronic oxidative, nitrosative, or sulfuric stress resulting from oxidant–antioxidant imbalance may be one of the causes of mitochondrial dysfunction and may consistently oxidize lipids, proteins, and DNA impairing their basic biological functions [50]. MDA formed in the reaction is one of the important markers of oxidative stress and the final product of lipid peroxidation. Strong oxidative stress leads to cell membrane damage and reflects the breakdown of polyunsaturated fatty acids (PUFA), hence it is an important element in the pathophysiology of schizophrenia [53,116]. PUFAs found in cell membranes are particularly vulnerable to oxidative damage. Therefore, the products of this process may contribute to the modification of physical properties of cell membranes. Evidence of this is the formation of excessive amount of MDA. In this paper, we draw attention to the existence of a negative correlation between MDA level and clinical status of schizophrenic patients in the course of psychotic decompensation, which is consistent with the results of Othmen et al. [117]. 

The aforementioned use of antipsychotic drugs, such as haloperidol, may contribute to the reduction of GSH activity by blocking the sigma 1 receptor. A decrease in the body’s natural antioxidant concentration may contribute to the increase of oxidative damage. Many studies show a negative correlation between the concentration of lipid peroxidation products and markers of antioxidative defense, indicating the important role of this process in the pathomechanism of schizophrenia [118,119]. In addition, GSH is synthesized from glutaminergic amino acids such as glycine, cysteine, and glutamic acid [120]. Decrease in glutamate levels, which is also observed in our study, can therefore lead to reduced GSH synthesis. In addition, the existing glutamate hypothesis, assuming the insufficient functioning of NMDA receptors, may explain the decreased glutamate neurotransmission [121]. GSH also enhances the NMDA receptor response to glutamate, which seems more to reflect a biological etiopathological role of psychosis mentioned the interrelationship of both metabolites [122]. In summary, the dysregulation of GSH level can be both the result of irregularities in the synthesis of its precursors and can be caused by pharmacotherapy. Both of these mechanisms deprive the body of its main antioxidant, making it more susceptible to free radical damage, which manifests itself by increasing the metabolites of oxidative stress in the periphery.

As for the observed changes in brain metabolites, the biggest differences between patients with schizophrenia, personality disorders, and controls, concerned levels of *N*-acetyl aspartate (NAA 2.02) and glutamate (GLN 2.45), although the effect was greatest for GLN. Patients with schizophrenia had significantly lower GLN levels compared to the control and personality disorder groups.

One of the mechanisms explaining lower levels of glutamate in the ACC region in people with schizophrenia during psychotic decompensation may be lower (compared to control) levels of vesicular glutamate transporters (vGLUT1) [123]. The percentage of patients with schizophrenia showing a similar profile of glutamate levels with cases of much lower concentrations of this metabolite in the ACC region was higher than in both controls. This may be associated with a specific predisposition in the schizophrenic population to a loss or significant reduction of vGLUT1, or with second-generation antipsychotic drugs [124]. However, the exact cause–effect relationship of the observed changes is difficult to establish.

Higher levels of NAA and GLN in the right and left frontal lobes predicted greater functional connectivity in the anterior frontal cortex of schizophrenics, while the opposite was true for ACC. These results suggest that in psychotic decompensation, the glutamate and *N*-acetyl aspartate (NAA) metabolism of the limbic system may play an important role in disrupting neuronal connectivity of this area. As reported by a small number of data mainly from post mortem studies, this may be associated with a characteristic neuronal pattern of this region of the brain in patients who exhibit abnormalities related to the response to treatment and thus may show resistance to it [123,125].

Interestingly, when linking the function of mitochondria with the changes in the brain depending on the treatment, attention should be paid to a multidirectional approach, as antipsychotics can have both positive and negative effects on mitochondria. Studies suggest that the changes in mitochondrial function in schizophrenia occur before treatment although they may also be the result of a psychotic process, and that the effects of treatment in schizophrenia causing impairment of mitochondrial function can be reversed [72].

People with paranoid schizophrenia obtained a lower average ratio of GLN/CR variables compared to the personality disorder and the control groups. The results obtained in this study are similar to those of Jelen et al., who noted a significant decrease in Glu/TCr (*p* = 0.004) and Glx/TCr (*p* < 0.001) compared to healthy controls [126], which may confirm a theory of glutaminergic neurotransmission deficits in schizophrenia. Abnormal increases in Glu/TCr and Glx/TCr during periods of relatively low executive load, without the same dynamic modulation as in healthy volunteers when solving tasks with increasing degrees of difficulty, suggest the underlying cause of glutaminergic neurotransmission abnormalities.

In assessing the clinical status of patients with schizophrenia, the presence of a positive correlation between the scale of positive symptoms (P on the PANSS scale) and GLU + GLN + GSH 3.7 in the ACC region also deserves attention. 

The observed decrease in levels of Glu, Glu/Cr, and GLU + GLN + GSH are strongly associated with the clinical state of the patients and depend on the phase of the disease in addition to the aforementioned mitochondrial function or the effect of the treatment. Higher Glu/Cr levels in ACC have been discussed in the literature, and are associated with greater severity of negative symptoms that may appear as a side effect after treatment when positive symptoms are muted but worsened.

In our study, it can be assumed that the obtained changes in the level of the brain metabolites may reflect the probable path of the disease development from complete physiology, through people with personality disorders as a precursor to the development of schizophrenia, to the breakdown of the compensatory mechanisms observed during psychotic decompensation as a deterioration of clinical symptoms. On the other hand, the observed effect may be related to the efficiency of the glutamate system and the function of mitochondria during the disease. On the other hand, it is a premise for further studies related to the assessment of the effectiveness of antipsychotic therapy in schizophrenia showing a specific similarity profile in the glutamate level in the ACC region. These studies can be used to further assess the effectiveness of the treatment based on the control of the efficiency of the glutaminergic system both in relation to positive symptoms as well as to the more and more difficult negative symptoms of the disease.

The limitation of this research are: older group of healthy volunteers compared to the patients, relatively low sensitivity of assessing the concentration of the brain metabolites, lack of reference to external standards, lack of functional assessment of changes in glutamate concentration using functional magnetic resonance spectroscopy (1H-fMRS) allowing dynamic monitoring of glutamate in activated/deactivated areas of the brain which would draw more accurate conclusions about the role of glutaminergic neurotransmission compared to standard magnetic resonance spectroscopy.

An additional limitation of this study could be the heterogeneity in terms of the differentiation (symptomatology) and severity of clinical symptoms (especially negative and/or cognitive) of the classified groups of patients.

## 5. Conclusions

Reduced levels of brain metabolites such as Glu, Gln, Cho, or NAA correlated with the parameters of peripheral oxidative stress and the deterioration of the clinical condition of people with schizophrenia. The observed increase in lactate and a decrease in pH of the brain in correlation with the increase in peripheral lipid peroxidation and decrease in total antioxidant potential were consistent with the impairment of the clinical condition of the patients and the observed neurocognitive deficits in the course of psychotic decompensation compared to people with personality disorders and healthy subjects. Our finding suggest a biological etiopathological role specific to psychosis, however, they need to be confirmed in larger studies so that further conclusions can be drawn from them. 

The levels of brain metabolites corresponding to given stages of the disease can be used to define the so-called schizotypes based on the selected biomarkers and, in the future, constitute the basis for targeted treatment for specific phenotypes of the disease.

Showing the differences between two psychiatric entities, such as schizophrenia and personality disorders, brings us closer to understanding the role of central markers and emphasizes their relationship with indicators of peripheral oxidative stress as potential markers responsible for the course of schizophrenia. It should also be remembered that personality disorders are a rapidly growing problem in modern psychiatry, thus focusing the attention of an increasing number of scientists who, through their research, try to fill the gap related to the insufficient number of comparative analyses between these two disease entities.

This study is an introduction to further research on clinical phenotypes of schizophrenia and the search for effective biomarkers improving the diagnosis of patients with psychosis compared to personality disorders and healthy controls. 

## Figures and Tables

**Table 1:** Sex and age of the respondents. Descriptive statistics of variables characteristic of the disease history for the F20, F60, and Control groups. Duration of untreated psychosis (DUP) and medical history data concerned the group of schizophrenics; DUP was counted from on the day of the onset of psychotic symptoms to the day of the patient’s first hospitalization due to psychosis. Abbreviations: M—mean; Me—median; SD—standard deviation; F20—paranoid schizophrenia in the course of psychotic decompensation according to the ICD 10 classification; F60—personality disorders according to ICD 10; n—number of patients; SD—standard deviation. Chi square test was used for sex, Kruskal–Wallis test—for age.

Variable		Group		
		F20	F60	Control
Sex; *n* (%)	Females	18 (45)	35 (85.4)	17 (56.7)
	Males	22 (55)	6 (14.6)	13 (43.3)
Age, mean (SD)		22.68 (7.39)	22.24 (8.43)	29.5 (5.33)
DUP (days)		24.3 (27.58)		
Hospitalization days		64.22 (33.16)		
Age of the first episode (years)		18.77 (3.75)		
Number of episodes		4 (4)		
Duration of illness (years)		4.02 (5.67)		

**Table 2:** Descriptive statistics on total antioxidant potential expressed as FRAP (ferric reducing ability of plasma) and malondialdehyde (MDA) in the compared groups of people. M—mean; Me—median; SD—standard deviation.

Variable	M			SD			Me			Statistical Result
	Control	F20	F60	Control	F20	F60	Control	F20	F60	
FRAP [mmol/L]	1.24	0.39	0.99	0.32	0.21	0.41	1.17	0.46	1.04	*γ*^2^(2) = 62.82; *p* < 0.001
MDA [mmol/L]	0.21	0.74	0.27	0.19	0.18	0.17	0.14	0.77	0.18	*γ*^2^(2) = 63.36; *p* < 0.001

**Table 3:** Differences between the groups in the range of metabolites (MRS results) in the ACC region of the brain. M—mean; Me—median; SD—standard deviation. LIP—lipids; LAC—lactate; ALA—alanine; NAA—N-Acetylaspartate; GLU—glucose; GABA—gamma-aminobutyric acid; GLN—glutamate; CR—creatine; CHO—choline; GLC—glucose; GLU + GLN + GSH—glucose/glutamate/glutathione; PCR + CR—phosphocreatine+creatine.

Variable	M			SD			Me			Statistical Result
	Control	F20	F60	Control	F20	F60	Control	F20	F60	
LIP 0.9–1.0	4,769,657	3,128,284.19	4,213,975.65	4923742.61	1,742,469.81	3,356,309.34	3,511,550	2,875,300	3,009,600	*γ*^2^(2) = 2.25; *p* = 0.32
LAC 1.33	3,986,255	3,590,129.03	5,347,930.43	1952562.47	2,290,871.91	3,373,001.8	3,807,050	2,926,600	4,250,700	*γ*^2^(2) = 5.39; *p* = 0.07
ALA 1.48	3,116,492.33	2,549,099.68	3,279,441.3	2250550.45	1,252,203.99	1,891,377.62	2,437,800	2,840,300	2,836,200	*γ*^2^(2) = 1.06; *p* = 0.59
NAA 2.02	35,592,666.67	21,412,329.03	26,459,343.48	49845895.22	8,148,541.71	7,783,049.3	25,970,000	23,198,000	25,634,000	*γ* *^2^* *(2) = 9.76;* *p = 0.008*
GLU 2.1	5,616,700	5,066,319.35	5,234,604.35	2145610.27	2,113,594.77	1,912,524.4	5,747,600	4,704,900	5,116,400	*γ*^2^(2) = 1.04; *p* = 0.6
GABA 2.3	11,950,866.67	7,924,832.26	7,804,804.35	23383742.18	4,695,692.97	4,519,643.27	7,798,100	6,683,600	6,726,100	*γ*^2^(2) = 0; *p* = 1
GLN 2.45	6,571,573.33	4,284,658.06	6,615,408.7	2911545.49	1,783,744.67	2,606,322.85	6,351,250	4,393,100	6,299,100	*γ* *^2^* *(2) = 18.33;* *p < 0.001*
CR 3.02	14,685,860	12,870,674.19	14,249,678.26	3213642.94	3,512,585.88	4,255,179.66	13,957,500	12,954,000	14,175,000	*γ*^2^(2) = 4.56; *p* = 0.1
CHO 3.22	11,826,310	10,704,625.81	12,965,773.91	4451263.58	2,963,926.23	5,022,070.96	11,776,000	10,469,000	1,283,1000	*γ*^2^(2) = 3.99; *p* = 0.14
GLC 3.43	4,469,010	3,242,659.03	5,493,995.65	2340956.02	1,348,296.84	3,839,775.13	3,975,650	3,187,100	4,424,400	*γ*^2^(2) = 5.69; *p* = 0.06
GLU + GLN + GSH 3.7	6,474,940	6,456,406.45	7,009,717.39	3,023,675.18	2,654,497.89	2,769,735.64	6,281,600	6,603,700	7,919,500	*γ*^2^(2) = 0.58; *p* = 0.75
GLC 3.8	7,791,786.67	5,946,009.68	6,798,052.17	3,690,099.48	2,795,699.54	3,126,442.87	7,434,450	5,737,500	5,663,300	*γ*^2^(2) = 0.48; *p* = 0.09
PCR + CR 3.9	12,587,493.33	10,337,651.61	16,627,239.13	5,680,273.03	5,179,535.32	26,591,342.08	12,107,000	9,643,300	12,864,000	*γ*^2^(2) = 2.51; *p* = 0.29

**Table 4:** Differences between the groups in the ratio of studied metabolites in the ACC area. M—mean; Me—median; SD—standard deviation; LIP/CR—lipids/creatine ratio; LAC/CR—lactate/creatine ratio; ALA/CR—alanine/creatine ratio; NAA/CR—N-Acetylaspartate/creatine ratio; GLU/CR—glucose/creatine ratio; GABA/CR—gamma-aminobutyric acid/creatine ratio; GLN/CR—glutamate/creatine ratio; CHO/CR—choline/creatine ratio; GLC/CR—glucose/creatine ratio; GLU+GLN+GSH/CR—glucose/glutamate/glutathione ratio.

Variable	M			SD			Me			Statistical Result
	Control	F20	F60	Control	F20	F60	Control	F20	F60	
LIP/CR	0.33	0.26	0.3	0.36	0.14	0.22	0.25	0.22	0.23	*γ*^2^(2) = 0.45; *p* = 0.8
LAC/CR	0.28	0.29	0.38	0.14	0.16	0.21	0.24	0.24	0.37	*γ*^2^(2) = 3.17; *p* = 0.21
ALA/CR	0.21	0.21	0.23	0.13	0.12	0.13	0.16	0.2	0.22	*γ*^2^(2) = 0.86; *p* = 0.65
NAA/CR	2.52	1.67	1.89	3.85	0.55	0.43	1.82	1.76	1.91	*γ*^2^(2) = 2.56; *p* = 0.28
GLU/CR	0.39	0.39	0.4	0.13	0.17	0.17	0.43	0.37	0.38	*γ*^2^(2) = 0.05; *p* = 0.98
GABA/CR	0.73	0.67	0.55	1.1	0.41	0.27	0.52	0.57	0.51	*γ*^2^(2) = 0.89; *p* = 0.64
GLN/CR	0.45	0.34	0.48	0.18	0.14	0.21	0.43	0.34	0.45	*γ* *^2^* *(2) = 9.17; p = 0.01*
CHO/CR	0.8	0.86	0.93	0.24	0.21	0.31	0.78	0.84	0.98	*γ*^2^(2) = 3.89; *p* = 0.16
GLC/CR	0.31	0.26	0.41	0.16	0.11	0.34	0.29	0.26	0.29	*γ*^2^(2) = 2; *p* = 0.37
GLU + GLN + GSH/CR	0.45	0.5	0.51	0.23	0.18	0.2	0.38	0.5	0.52	*γ*^2^(2) = 1.69; *p* = 0.43
GLC/CR	0.52	0.45	0.48	0.19	0.24	0.18	0.53	0.4	0.42	*γ*^2^(2) = 4.26; *p* = 0.12

**Table 5:** Statistically significant predictors of regression analysis for the dependent variable (quality of life). CR—creatine; LAC—lactate; NAA—*N*-Acetylaspartate; GLN—glutamate; CHO—choline; GLC—glucose; PCR + CR—phosphocreatine + creatine.

Predictor	Beta	Statistical Significance
GLC 3.8 (right frontal lobe)	0.37	0.02
PCR + CR 3.9 (right frontal lobe)	−0.49	0.001
LAC 1.33 (left frontal lobe)	−0.44	0.006
NAA 2.02 (left frontal lobe)	0.36	0.03
GLN 2.45 (left frontal lobe)	−0.62	<0.001
GLC 3.8 (left frontal lobe)	0.43	0.007

## Supplementary Materials

The following are available online at https://www.mdpi.com/2218-273X/10/9/1272/s1. Table S1. The relationship between the quality of life of the examined people and the analyzed brain metabolites.; Table S2. The relationship between the quality of life and the ratios of the analyzed brain parameters.; Table S3. The relationship between FRAP and MDA levels, and biochemical parameters in the compared groups of people.; Table S4. The relationship between FRAP and MDA levels, and the ratios of biochemical parameters in the compared groups of people.
