# Supplementary material for: The Relationship between the Level of Anterior Cingulate Cortex Metabolites, Brain-Periphery Redox Imbalance, and the Clinical State of Patients with Schizophrenia and Personality Disorders
Source: Biomolecules. 2020 Sep 3;10(9):1272. doi: 10.3390/biom10091272 (PMC7565827; doi:10.3390/biom10091272)
Supplement: Supplementary file 1 [file biomolecules-10-01272-s001.pdf]

Supplementary Materials:

Table S1. The relationship between the quality of life of the examined people and the analyzed brain metabolites.

Variable		P scale	N scale	G scale	T scale
LIP 0.9-1.0	Right frontal lobe	0.08	-0.04	0.09	0.06
	Left frontal lobe	-0.02	-0.03	0.03	-0.01
	ACC	-0.03	-0.25	-0.03	-0.01
LAC 1.33	Right frontal lobe	-0.15	-0.17	-0.09	-0.09
	Left frontal lobe	-0.09	-0.31	-0.11	-0.08
	ACC	0.01	0.02	-0.09	-0.14
ALA 1.48	Right frontal lobe	0.16	0.2	0.13	0.14
	Left frontal lobe	-0.1	-0.17	0.09	-0.03
	ACC	-0.04	-0.17	-0.12	-0.16
NAA 2.02	Right frontal lobe	-0.15	-0.16	-0.09	-0.09
	Left frontal lobe	0.15	0.02	0.1	0.23
	ACC	0.17	-0.15	-0.11	-0.07
GLU 2.1	Right frontal lobe	-0.2	-0.2	-0.04	-0.11
	Left frontal lobe	-0.07	-0.16	-0.08	-0.09
	ACC	-0.27	-0.33	***-0.37; p = 0.04***	***-0.36; p = 0.04***
GABA 2.3	Right frontal lobe	0.03	0	0.11	0.09
	Left frontal lobe	0.15	0.23	0.11	0.19
	ACC	0.22	0.07	0.26	0.25
GLN 2.45	Right frontal lobe	0.11	0.05	0.29	0.16
	Left frontal lobe	-0.13	-0.25	-0.13	-0.19
	ACC	0.12	0.14	0.14	0.08
CR 3.02	Right frontal lobe	-0.25	-0.22	-0.32	-0.23
	Left frontal lobe	0.11	-0.17	0.03	0.09
	ACC	0.16	-0.06	-0.01	0.03
CHO 3.22	Right frontal lobe	-0.07	-0.13	-0.02	-0.05
	Left frontal lobe	0.04	-0.09	0.02	0.05
	ACC	0.31	0.11	-0.02	0.13
GLC 3.43	Right frontal lobe	-0.18	-0.08	-0.21	-0.15
	Left frontal lobe	0.04	-0.04	0	0.03
	ACC	-0.07	0.02	-0.31	-0.13
GLU+GLN+GSH 3.7	Right frontal lobe	-0.02	-0.04	0.1	0
	Left frontal lobe	-0.01	-0.02	0.06	0.01
	ACC	***0.38; p = 0.04***	0.05	0.27	0.21
GLC 3.8	Right frontal lobe	-0.19	-0.03	-0.09	-0.08
	Left frontal lobe	0.09	-0.14	-0.09	-0.05
	ACC	-0.16	-0.26	-0.14	-0.18
PCR+CR 3.9	Right frontal lobe	-0.24	-0.13	-0.35	-0.25
	Left frontal lobe	-0.17	***-0.37; p = 0.04***	-0.23	-0.26
	ACC	0.02	-0.2	-0.12	-0.02

LIP – lipids; LAC – lactate; ALA – alanine; NAA - N-Acetylaspartate; GLU – glucose; GABA - gamma-aminobutyric acid; GLN – glutamate; CR – creatine; CHO – choline; GLC - glucose; GLU+GLN+GSH

– glucose/glutamate/glutathione; PCR+CR – phosphocreatine+creatine; ACC – anterior cingulate cortex.

Table S2. The relationship between the quality of life and the ratios of the analyzed brain parameters.

**Variable**		**P scale**	**N scale**	**G scale**	**T scale**
LIP/CR	Right frontal lobe	0.11	-0.01	0.14	0.1
	Left frontal lobe	-0.05	0	0.01	-0.04
	ACC	-0.1	-0.28	-0.03	0
LAC/CR	Right frontal lobe	-0.03	-0.06	0.08	0.09
	Left frontal lobe	-0.08	-0.19	-0.07	-0.07
	ACC	0.02	0.11	0.03	-0.09
ALA/CR	Right frontal lobe	0.23	0.22	0.23	0.2
	Left frontal lobe	-0.16	-0.14	0.08	-0.08
	ACC	-0.02	0.02	0.04	-0.05
NAA/CR	Right frontal lobe	0.12	0.07	0.21	0.19
	Left frontal lobe	0.18	0.27	0.16	0.25
	ACC	0.19	-0.06	-0.01	0.07
GLU/CR	Right frontal lobe	-0.06	-0.03	0.14	0.04
	Left frontal lobe	-0.09	-0.05	-0.03	-0.08
	ACC	-0.34	-0.22	-0.31	-0.29
GABA/CR	Right frontal lobe	0.08	-0.01	0.16	0.11
	Left frontal lobe	0.12	0.33	0.12	0.18
	ACC	0.15	0.02	0.28	0.25
GLN/CR	Right frontal lobe	0.17	0.2	***0.41; p = 0.02***	0.29
	Left frontal lobe	-0.14	-0.17	-0.1	-0.24
	ACC	0.06	0.25	0.24	0.13
CHO/CR	Right frontal lobe	0.28	0.21	***0.38; p = 0.03***	0.3
	Left frontal lobe	0	0.08	0.01	0.02
	ACC	0.14	0.26	0.02	0.19
GLC/MR	Right frontal lobe	-0.08	0.03	-0.06	-0.02
	Left frontal lobe	0.01	0.03	0.01	0.01
	ACC	-0.12	-0.18	-0.14	-0.13
GLU+GLN+GSH/CR	Right frontal lobe	-0.03	-0.02	0.11	-0.04
	Left frontal lobe	-0.04	0.13	0.07	0.01
	ACC	0.36	0.04	0.33	0.25
GLC/CR	Right frontal lobe	-0.08	0.03	-0.06	-0.02
	Left frontal lobe	-0.07	0.12	-0.29	-0.14
	ACC	-0.27	***-0.46; p = 0.01***	-0.1	-0.23

LIP/CR – lipids/creatine ratio; LAC/CR – lactate/creatine ratio; ALA/CR – alanine/creatine ratio; NAA/CR - N-Acetylaspartate/creatine ratio; GLU/CR – glucose/creatine ratio; GABA/CR - gamma-aminobutyric acid/creatine ratio; GLN/CR – glutamate/creatine ratio; CHO/CR – choline/creatine ratio; GLC/CR - glucose/creatine ratio; GLU+GLN+GSH/CR – glucose/glutamate/glutathione ratio; ACC – anterior cingulate cortex.

Table S3. The relationship between FRAP and MDA levels, and biochemical parameters in the compared groups of people.

Variable		Group					
		F20		F60		Control	
		FRAP	MDA	FRAP	MDA	FRAP	MDA
LIP 0.9-1.0	Right frontal lobe	0.07	0.02	-0.1	-0.01	-0.28	-0.12
	Left frontal lobe	-0.26	0.11	-0.1	0.23	0.13	-0.08
	ACC	-0.28	-0.06	***-0.58; p = 0.004***	0.24	-0.17	-0.26
LAC 1.33	Right frontal lobe	-0.06	-0.01	0.14	0	-0.12	-0.21
	Left frontal lobe	-0.05	-0.27	0.02	0.07	-0.29	-0.07
	ACC	0.1	-0.16	-0.14	0.27	0.01	-0.2
ALA 1.48	Right frontal lobe	0.17	-0.26	0.12	-0.02	-0.27	0.01
	Left frontal lobe	0.02	0.03	***0.43; p = 0.04***	***-0.53; p = 0.009***	-0.37	-0.08
	ACC	***-0.43; p = 0.02***	0	-0.17	-0.04	-0.05	-0.02
NAA 2.02	Right frontal lobe	-0.17	0.15	-0.07	0.01	-0.12	0.32
	Left frontal lobe	-0.07	0.17	0.07	-0.4	0.22	0.29
	ACC	-0.11	-0.24	-0.4	0.02	***0.41; p = 0.02***	-0.19
GLU 2.1	Right frontal lobe	0.03	0.08	-0.33	***0.57; p = 0.004***	-0.15	0.04
	Left frontal lobe	***-0.52; p - 0.002***	0.26	0.08	-0.29	0.11	0.19
	ACC	-0.13	-0.23	0.01	0.35	***0.5; p = 0.005***	***0.52; p = 0.004***
GABA 2.3	Right frontal lobe	0.09	0.09	-0.06	0.02	0.02	0.16
	Left frontal lobe	-0.12	0.06	-0.2	-0.06	0.09	-0.09
	ACC	0.16	-0.31	-0.37	0.26	0.07	-0.04
GLN 2.45	Right frontal lobe	0.02	-0.26	0.04	-0.04	0.16	-0.12
	Left frontal lobe	0.04	0.04	0.04	-0.17	-0.07	0.09

	ACC	***0.43; p = 0.02***	-0.32	-0.27	0.15	0.31	0.12
CR 3.02	Right frontal lobe	-0.2	-0.08	-0.25	0.23	0	0.13
	Left frontal lobe	-0.05	0.17	0.1	-0.18	0.33	0.38
	ACC	-0.2	***-0.4; p = 0.03***	-0.38	0.13	0.14	-0.24
CHO 3.22	Right frontal lobe	0.05	-0.06	0.17	0	-0.01	0.12
	Left frontal lobe	0.33	0.04	0.12	-0.09	-0.14	0.14
	ACC	-0.05	-0.25	-0.19	-0.03	0.2	-0.25
GLC 3.43	Right frontal lobe	-0.05	-0.16	0.21	-0.13	-0.36	0.21
	Left frontal lobe	-0.23	0	0.28	-0.26	0.06	0.18
	ACC	-0.03	-0.02	0.04	0.08	0.22	-0.15
GLU+GLN+GSH 3.7	Right frontal lobe	0.2	-0.04	-0.41	***0.51; p = 0.01***	-0.17	0.36
	Left frontal lobe	0	0.24	-0.36	0.17	-0.07	0.04
	ACC	-0.05	***-0.42; p = 0.02***	-0.09	-0.07	***0.46; p = 0.01***	-0.13
GLC 3.8	Right frontal lobe	0.09	0.26	0.13	-0.18	0.13	-0.04
	Left frontal lobe	-0.06	-0.3	-0.17	-0.15	0.26	0.14
	ACC	0.24	-0.35	***-0.61; p = 0.002***	0.35	0.14	-0.03
PCR+CR 3.9	Right frontal lobe	-0.05	0.12	-0.31	0.24	0.12	0.02
	Left frontal lobe	0	-0.17	-0.07	-0.21	-0.26	-0.23
	ACC	0.04	-0.17	-0.04	-0.29	0.28	-0.05

LIP – lipids; LAC – lactate; ALA – alanine; NAA - N-Acetylaspartate; GLU – glucose; GABA - gamma-aminobutyric acid; GLN – glutamate; CR – creatine; CHO – choline; GLC - glucose; GLU+GLN+GSH – glucose/glutamate/glutathione; PCR+CR – phosphocreatine+creatine; ACC – anterior cingulate cortex.

Table S4. The relationship between FRAP and MDA levels, and the ratios of biochemical parameters in the compared groups of people.

Variable		Group					
		F20		F60		Control	
		FRAP	MDA	FRAP	MDA	FRAP	MDA
LIP/CR	Right frontal lobe	0.11	0.04	0.06	-0.01	-0.37	-0.08
	Left frontal lobe	-0.26	0.08	-0.19	***0.42; p = 0.007***	0	-0.2
	ACC	-0.16	0.05	***-0.5; p = 0.02***	0.21	-0.26	-0.14
LA/CR	Right frontal lobe	0.01	0.06	0.29	-0.14	-0.22	-0.21
	Left frontal lobe	-0.02	-0.33	-0.05	0.25	***-0.41; p = 0.03***	-0.16
	ACC	0.15	-0.1	0.03	0.15	-0.03	-0.14
ALA/CR	Right frontal lobe	0.17	-0.28	0.15	-0.09	-0.32	0.03
	Left frontal lobe	0.11	-0.04	***0.43; p = 0.04***	***-0.43; p = 0.04***	***-0.44; p = 0.01***	-0.22
	ACC	-0.37	0.2	-0.04	-0.01	0	0.1
NAA/CR	Right frontal lobe	0.05	0.27	0.24	-0.22	-0.11	0.19
	Left frontal lobe	0.04	-0.08	0.02	-0.13	-0.2	-0.13
	ACC	0	-0.04	0.09	-0.16	0.38	0.04
GLU/CR	Right frontal lobe	0.18	0.07	-0.22	***0.57; p = 0.004***	-0.22	-0.01
	Left frontal lobe	***-0.41; p = 0.02***	0.25	0.01	-0.1	-0.06	-0.06
	ACC	-0.08	-0.01	0.21	0.14	***0.46; p = 0.01***	***0.59; p = 0.001***
GABA/CR	Right frontal lobe	0.14	0.09	0.14	-0.11	-0.04	0.2
	Left frontal lobe	-0.05	-0.02	-0.31	0.07	-0.13	-0.3
	ACC	0.26	-0.21	-0.26	0.24	0.01	0.04
GLN/CR	Right frontal lobe	0.06	-0.12	0.16	-0.1	0.01	-0.24
	Left frontal lobe	0.26	-0.19	-0.16	0.12	-0.36	-0.2
	ACC	***0.62; p = 0.001***	-0.08	-0.12	0.09	0.34	0.27

CHO/CR	Right frontal lobe	0.25	-0.04	***0.47; p = 0.02***	-0.17	-0.16	0.02
	Left frontal lobe	0.37	-0.09	-0.09	0.13	***-0.43; p = 0.02***	-0.21
	ACC	0.23	0.01	0.13	-0.2	0.13	-0.15
GLC/MR	Right frontal lobe	0.01	-0.11	0.28	-0.19	-0.36	0.08
	Left frontal lobe	-0.19	-0.03	0.25	-0.22	-0.09	0
	ACC	0.24	0.23	0.2	0	0.15	-0.12
GLU+GLN +GSH/CR	Right frontal lobe	0.28	-0.04	-0.15	0.41	-0.13	0.35
	Left frontal lobe	0.09	0.12	-0.31	0.2	-0.19	-0.08
	ACC	0.01	-0.15	0.21	-0.2	0.38	-0.12
GLC/CR	Right frontal lobe	0.01	-0.11	0.28	-0.19	-0.36	0.08
	Left frontal lobe	-0.06	***-0.39; p = 0.03***	-0.31	0.07	0.14	0.04
	ACC	0.24	-0.11	***-0.54; p = 0.008***	0.39	0.2	0.1

LIP/CR – lipids/creatine ratio; LAC/CR – lactate/creatine ratio; ALA/CR – alanine/creatine ratio; NAA/CR - N-Acetylaspartate/creatine ratio; GLU/CR – glucose/creatine ratio; GABA/CR - gamma-aminobutyric acid/creatine ratio; GLN/CR – glutamate/creatine ratio; CHO/CR – choline/creatine ratio; GLC/CR - glucose/creatine ratio; GLU+GLN+GSH/CR – glucose/glutamate/glutathione ratio; ACC – anterior cingulate cortex.
